# Supplementary material for: Microbiome depletion and recovery in the sea anemone, Exaiptasia diaphana, following antibiotic exposure
Source: mSystems. 2024 May 17;9(6):e01342-23. doi: 10.1128/msystems.01342-23 (PMC11237641; doi:10.1128/msystems.01342-23)
Supplement: Supplemental Information — Legends for supplemental tables. [file msystems.01342-23-s0001.docx]

**Microbiome depletion and recovery in the sea anemone, *Exaiptasia diaphana*, following antibiotic exposure**

Sophie MacVittie^a^, Saam Doroodian^a^, Aaron Alberto^a^, Maggie Sogin^a*^

^a^Department of Molecular Cell Biology, University of California, Merced, California, USA

Running Head: Antibiotic treatment reduces the Aiptasia microbiome

*Corresponding Author: [esogin@ucmerced.edu](mailto:esogin@ucmerced.edu)

**Supplemental Information**

### **Table S1.** Model statistics for treatment comparisons across physiological and microbiome variables.

**Table S2.** Means and standard error for physiological variables across experimental treatments.

**Table S3.** Members of the Aiptasia core microbiome.

**Table S4.** Differentially abundant taxa and patterns of removal.
